# *PpMYB4*-mediated regulation of wax accumulation and abiotic stress responses in Kentucky bluegrass (*Poa pratensis* L.)

**DOI:** 10.3389/fpls.2026.1811600

**Published:** 2026-05-13

**Authors:** Yifeng Jin, Xue You, Xiaoxue Liang, Yuwen Wang, Qi Zhen, Xinyu Liu, Yang Chen, Miao He

**Affiliations:** 1 College of Landscape Architecture, Northeast Forestry University, Harbin, China; 2 College of Life Science and Agriculture Forestry, Qiqihar University, Qiqihar, China; 3 College of Chemistry, Chemical Engineering and Resource Utilization, Northeast Forestry University, Harbin, China; 4 Heilongjiang Province Key Laboratory of Resistance Gene Engineering and Preservation of Biodiversity in Cold Areas, Qiqihar, Heilongjiang, China

**Keywords:** abiotic stress, expression analysis, Kentucky bluegrass, *PpMYB4*, transcription factor

## Abstract

Drought is a primary factor constraining the growth and development of Kentucky bluegrass (Poa pratensis L.). To elucidate the molecular mechanisms underlying drought and saline–alkali stress responses, the cultivar ‘K.B.G’ was used as the experimental material. An R2R3-MYB transcription factor gene, PpMYB4, and its promoter were cloned and functionally characterized. Subcellular localization analysis, confirmed by DAPI co-staining, demonstrated that PpMYB4 is targeted to the nucleus, consistent with its role as a transcription factor. Phylogenetic analysis revealed the closest relationships with MYB4 homologs from Brachypodium distachyon, Lolium rigidum, and wheat (Triticum aestivum). RT-qPCR showed that PpMYB4 is preferentially expressed in stems and is transcriptionally responsive to drought, saline-alkali stress, and multiple phytohormones (SA, IAA, MeJA induced; ABA, GA repressed). Overexpression of PpMYB4 in Kentucky bluegrass, confirmed by PCR-based transgene detection and qRT-PCR, promoted vegetative growth, enhanced rooting, and increased epicuticular wax deposition on leaf surfaces. Under drought stress, overexpression lines maintained higher relative water content, lower electrolyte leakage, and elevated SOD and POD activities compared with wild-type plants. Promoter deletion analysis identified an upstream region critical for transcriptional activity and demonstrated drought-inducible promoter function. These findings establish PpMYB4 as a multifunctional regulator of wax accumulation and antioxidant defense in Kentucky bluegrass, providing a candidate gene for molecular breeding of drought-resilient turfgrass.

## 1 Introduction

Urban lawns have been recognized as fundamental components of green spaces and have been closely associated with daily life and urban landscape formation (Ignatieva et al., 2017). Globally, turfgrasses have been extensively cultivated, through which substantial economic value and ecological benefits have been provided (Zhao et al., 2015). Kentucky bluegrass (*Poa pratensis* L.), a perennial species within Poaceae, has been widely used as one of the major cool-season turfgrasses (Fu et al., 2007). Because well-developed rhizomes have been formed, this turfgrass has been extensively used in sports fields, golf-course roughs, residential lawns, roadside greening, and public parks (Bushman et al., 2016). Despite its excellent quality, limited drought tolerance has been reported for Kentucky bluegrass, and growth cessation has been observed under drought conditions (Yu et al., 2025). Drought stress, a major abiotic constraint on plant development, has been shown to inhibit growth through root dehydration and stomatal closure, thereby ultimately reducing photosynthetic rate (Hou et al., 2014). Therefore, in-depth investigation of drought tolerance in Kentucky bluegrass has been required to facilitate stress-resilient breeding and improve ecological adaptability of turfgrass.

Transcription factors have been encoded by regulatory genes and have been activated by diverse signals. Accordingly, pivotal roles in plant stress-response systems have been executed via regulation of downstream stress-responsive genes (Wang et al., 2021b). Among transcription-factor families, the MYB family has been regarded as one of the largest and most diverse, and broad distribution across eukaryotes has been documented (Wang et al., 2023b). MYB transcription factors have been characterized by binding to MYB-binding sites (MBS) within target promoters; thus, regulation of hormone responses (including abscisic acid, ABA, and gibberellins, GA) and modulation of abiotic-stress responses (e.g., salinity and drought) have been mediated. Consequently, plant stress tolerance has been enhanced (He et al., 2012). On the basis of MYB-repeat composition, MYB transcription factors have been categorized into 1R-MYB, R2R3-MYB, 3R-MYB, and 4R-MYB types (Katiyar et al., 2012; Li et al., 2022), among them, MYB4 (an R2R3-MYB) has been increasingly investigated in plant functional genomics and crop improvement because diverse biological functions have been implicated (Aydin et al., 2012; Agarwal et al., 2020).

In recent years, an increasing number of *MYB4* genes have been cloned, and functions of several *MYB4* homologs have been elucidated. For example, distinct functions have been reported for *Arabidopsis AtMYB4* (Wang et al., 2020), grape (*Vitis vinifera*) *VvMYB4-like* (Pérez-Díaz et al., 2016), and tobacco (*Nicotiana tabacum*) *NtMYB4* (Luo et al., 2020), including inhibition of phenylalanine synthesis, suppression of anthocyanin accumulation, and positive regulation of anthocyanin biosynthesis, respectively. In *Arabidopsis*, inhibition of flavonoid biosynthesis has been attributed to *MYB4*-mediated disruption of MBW-complex transcriptional activity and concomitant downregulation of *ADT6* expression (Allan et al., 2008). Beyond flavonoid metabolism, involvement of *MYB4* in lignin-biosynthesis regulation has also been reported. For instance, diminished lignification has been observed when wheat (*Triticum aestivum*) *TaMYB4* has been heterologously expressed in tobacco (Ma et al., 2011).

Under abiotic stress, proline accumulation has been promoted and drought resistance has been enhanced when *OsMYB4* (rice, *Oryza sativa* L.) and *PgMYB4* (ginseng, *Panax ginseng*) have been ectopically expressed in *Arabidopsis* (Lian et al., 2021). Under salt stress, significant growth advantages have been observed in tobacco in which *LpMYB4* from *Lilium pumilum* has been overexpressed, as reduced leaf wilting and lodging have been detected relative to wild-type tobacco. Furthermore, physiological measurements have supported that plant tolerance to salt stress has been enhanced by heterologous expression of *LpMYB4* (Zhang et al., 2024a). In addition, under cadmium stress, expression of plant chelating peptide synthesis genes (*PCS1* and *MT1C*) has been activated by *MYB4*; antioxidant defenses have been synergistically enhanced, and cadmium tolerance has thus been positively regulated in *Arabidopsis* (Agarwal et al., 2020). Collectively, *MYB4* has been implicated in mediating plant growth, development, and responses to multiple abiotic stresses.

Notably, the MYB family, a large and functionally diverse group of transcription factors in plants, has been extensively validated as a key regulator of abiotic-stress responses across diverse species (Lv et al., 2023; Chen et al., 2018; Zhou et al., 2023; Zhang et al., 2023b). Although functions of MYB transcription factors have been widely investigated in cereal crops such as rice (Huang et al., 2015) and maize (*Zea mays* L.) (Ren et al., 2024), systematic elucidation of the mechanisms by which MYB members respond to abiotic stress has remained limited in turfgrasses, particularly Kentucky bluegrass. Accordingly, Kentucky bluegrass was used for cloning of *PpMYB4* and its promoter; expression patterns were analyzed across different tissues, under abiotic stresses, and following hormone treatments, and promoter activity was evaluated. The expression basis and putative regulatory network of *PpMYB4* in Kentucky bluegrass stress responses were intended to be elucidated; thereby, evidence for deeper understanding of the molecular mechanisms underlying stress responses was to be provided, and a foundation for genetic improvement of turfgrass stress resistance through *PpMYB4* utilization was to be established.

## 2 Materials and methods

### 2.1 Plant materials and growing conditions

Kentucky bluegrass was used as the experimental material. After screening, seeds were evenly sown in a cultivation medium composed of soil, sand, and vermiculite at a 3:1:1 ratio. Growth conditions were maintained at 25 °C/15°C (day/night), 60% relative humidity, a 14-h photoperiod, and a light intensity of 600 µmol m^-2^s^-1^. After a 2-month acclimation period under controlled conditions, plants were sampled and subsequently subjected to stress treatments.

### 2.2 Cloning of *PpMYB4* gene

Total RNA was extracted from Kentucky bluegrass using a plant RNA extraction kit (TIANGEN, China). First-strand cDNA was synthesized by reverse transcription using the HiFiScript cDNA Synthesis Kit (CWBIO, China). Based on sequencing information, gene-specific primers (**Supplementary File 1)** were designed; the ORF (open reading frame) of PpMYB4 was amplified, and the PCR products were sequenced.

### 2.3 *PpMYB4* bioainformatics analysis

For phylogenetic and motif analyses, MEME (https://meme-suite.org/meme) and TBtools-II (v2.323) were used. Multiple sequence alignment was performed using ClustalOmega (https://www.ebi.ac.uk/jdispatcher/msa/clustalo) to generate aligned sequences for downstream analyses. Subsequently, subcellular localization was predicted using Plant mPloc (http://www.csbio.sjtu.edu.cn/bioinf/plant-multi) to support functional inference at the cellular level.

### 2.4 Subcellular localization analysis of *PpMYB4*

The pCAMBIA1300 vector plasmid was extracted, and both pCAMBIA1300 and pMD18T-*MYB4* plasmids were subjected to double-enzyme digestion to recover and purify the target fragment. Subsequently, *PpMYB4* was inserted into the pCAMBIA1300-EGFP vector via homologous recombination, and the recombinant vector pCAMBIA1300-*PpMYB4*-EGFP was constructed. The ligation product was transformed into competent *Escherichia coli* cells, and positive clones were screened. The validated recombinant plasmid was then introduced into *Agrobacterium tumefaciens* competent cells (GV3101) and confirmed by PCR. *Nicotiana benthamiana* plants were used as transient-expression hosts. Using syringe infiltration, *A. tumefaciens* harboring the pCAMBIA1300-*PpMYB4*-EGFP recombinant plasmid was introduced into *N. benthamiana* leaves. After infiltration, plants were incubated in darkness for 24 h and subsequently transferred to light for an additional 24 h. To confirm nuclear localization, infiltrated leaf segments were stained with 4’, 6-diamidino-2-phenylindole (DAPI; 1 µg/mL) for 10 min in the dark, rinsed with PBS, and immediately examined. Samples were observed using a confocal laser scanning microscope (Zeiss LSM 880); GFP excitation was set at 488 nm (emission 500–550 nm), DAPI excitation at 405 nm (emission 420–480 nm), and chloroplast autofluorescence at 633 nm (emission 650–750 nm). Fluorescence images were merged to assess co-localization.

### 2.5 Expression pattern analysis of the *PpMYB4* gene in Kentucky bluegrass

Expression patterns of *PpMYB4* were analyzed across cultivars and tissues and under abiotic stresses and hormone treatments in Kentucky bluegrass.Three cultivars (K.B.G, Arcadia, and Jenny) were selected. Root, stem, and leaf tissues were collected from healthy, mature K.B.G plants. Roots were washed and plants were transferred to 1/2 Hoagland nutrient solution for 14 days, after which abiotic-stress treatments were applied. After sampling, all tissues were immediately frozen in liquid nitrogen and stored at -80 °C for subsequent analyses of stress-induced expression. Stress treatments were conducted as follows: (1) drought stress, for which a natural dehydration method was used to simulate field drought conditions, and leaves were sampled at 0, 3, 7, and 10 days (Yu et al., 2025). (2) saline–alkali stress, for which mixed solutions of NaCl, Na_2_SO_4_, NaHCO_3_, and Na_2_CO_3_ were prepared at 50, 100, and 150 mM; roots were irrigated regularly, and leaves were sampled after 3 days (Bushman et al., 2016; Zhang et al., 2024a). For hormone treatments, abscisic acid (ABA; 30 mg/L), salicylic acid (SA; 200 mg/L), indole-3-acetic acid (IAA; 800 mg/L), gibberellin (GA; 200 mg/L), and methyl jasmonate (MeJA; 50 mg/L) were applied, and each treatment was conducted in separate pots (Wei et al., 2021; Li et al., 2021). Soil-grown healthy leaves were sprayed daily until droplets no longer adhered to the surface. After 5 days of hormone treatment, relative expression of *PpMYB4* was quantified. All experiments were performed with three biological replicates.

Total RNA was extracted from the collected samples and subsequently reverse-transcribed into cDNA. qRT-PCR was used for relative quantification of gene expression, and primer sequences are listed in **Supplementary File 1**. Three biological and three technical replicates were included, and relative expression levels were calculated using the 2^–∆∆Ct^ method (Schmittgen and Livak, 2008).

### 2.6 Transformation and characterization of *PpMYB4* overexpression Kentucky bluegrass

Referring to the methods of Sun et al. (2022), modifications were made using *A. tumefaciens* -mediated transformation to transform the Kentucky bluegrass variety “K.B.G”. Stem segments (1.5 cm) were excised from the basal region of sterile seedlings (8–10 weeks old) and inoculated onto differentiation medium for 1 day of pre-culture. After *A. tumefaciens* strain GV3101 harboring the recombinant vector pC1300S-*PpMYB4* had been activated, 20 mL of bacterial culture was collected by centrifugation at 4 °C and 4000 rpm for 15 min. Bacterial cells were resuspended in 1/2 MS liquid medium supplemented with 5% sucrose, and the suspension was adjusted to OD_600_ = 0.8. Subsequently, resistant seedlings were transferred to antibiotic-free medium or nutrient-deficient soil for cultivation.

Molecular identification of transgenic plants was performed as follows: Genomic DNA was extracted from leaves of hygromycin-resistant plants; wild-type (WT) DNA was used as a negative control, and Wild-type (WT) DNA was used as the negative control, and water was used as the blank control. The hygromycin phosphotransferase gene (*hpt*) fragment was amplified by PCR to validate transgenic positive plants. To confirm overexpression, total RNA was extracted from two independent PCR-positive T0 lines (OE-2, OE-4) and WT plants under non-stress conditions. qRT-PCR was performed using *PpMYB4*-specific primers with *PpUBQ* as the reference gene. Semi-quantitative RT-PCR was also conducted with 28 PCR cycles to visually confirm elevated *PpMYB4* transcript levels in OE lines relative to WT (**Supplementary File 1**).

### 2.7 Determination of chlorophyll content and phenotypic data in Kentucky bluegrass

Chlorophyll extraction was performed according to the protocol described in the previously published study (Wang et al., 2023a).Leaves (0.2 g) were soaked in 20 mL of 95% ethanol and were extracted in darkness until the tissue became white. Absorbance of the extract was measured at 645 nm and 663 nm. Chlorophyll content was calculated using the following formula: Chl=0.04×(20.21×D645+8.02×D663).

After 60 days of co-culture, the numbers of clustered shoots and branches were observed under a microscope. To minimize individual variation, ten independent explants were used, and mean values were calculated. In addition, ten plants were randomly selected, and plant height was measured from the stem base to the growth point using a ruler to calculate mean height. Overexpression plants and wild-type plants were grown under identical conditions, and rooting rate, differentiation rate, and growth rate were calculated and compared. Leaf scanning electron microscopy (SEM) was conducted according to the method described by Jiang et al. (2024). Fresh leaves from corresponding positions of overexpression and control plants were collected and examined by SEM to compare epidermal ultrastructure between *PpMYB4*-overexpressing Kentucky bluegrass and wild-type plants. After fixation, dehydration, critical-point drying, and ion-sputter coating (IXRF550i), samples were observed using a scanning electron microscope (HITACHI SU8010).

### 2.8 Cloning and bioinformatics analysis of *PpMYB4* promoter in Kentucky bluegrass

The complete Kentucky bluegrass genome (CAMZMU0100051.1) was queried to obtain the promoter sequence of *PpMYB4*. Gene-specific primers (*PpMYB4Qi*-F/R) were designed, and sequences are provided in **Supplementary File 1**. PCR amplification was performed using gDNA derived from Kentucky bluegrass ‘K.B.G’ as the template, with *PpMYB4Qi*-F/R used as forward and reverse primers. After the *PpMYB4* promoter sequence had been obtained, cis-acting elements were predicted and functionally annotated using PlantCARE (https://bioinformatics.psb.ugent.be/webtools/plantcare/html), and results were visualized using TBtools (Simple BioSequence Viewer).

### 2.9 Activity analysis of the *PpMYB4* promoter in Kentucky bluegrass

To investigate the transcriptional regulatory mechanism of *PpMYB4*, promoter activity was analyzed using *A. tumefaciens*-mediated transient transformation. The full-length *PpMYB4* promoter (GUS-MYB4) and three 5’-end deletion fragments (F1/R, F2/R, and F3/R) were fused to the GUS reporter to construct plant expression vectors, which were introduced into *N. benthamiana* leaves. Untreated leaves (CK) and the empty vector (pBI121) were used as negative controls, while the vector containing the promoter CaMV 35S served as a positive control.

## 3 Results

### 3.1 Cloning of *PpMYB4* gene

Full-length amplification of the *PpMYB4* coding sequence was performed using cDNA from Kentucky bluegrass. A single, distinct PCR product of 827 bp was obtained (**Supplementary File 1**), indicating successful amplification of the target transcript. The amplicon was subsequently recovered, cloned into a TA cloning vector, and verified by Sanger sequencing. Sequence validation confirmed that the cloned fragment corresponded to the *PpMYB4* open reading frame and showed high sequence integrity with no unexpected insertions or deletions.

To further characterize the cloned gene at the protein level, the deduced amino acid sequence was analyzed for conserved domains. Domain annotation indicated that the encoded protein contained the characteristic conserved region of the PLN03091 superfamily (**Supplementary File 1**), which is consistent with typical features of plant MYB transcription factors. Collectively, these results demonstrated that *PpMYB4* was successfully cloned from Kentucky bluegrass and that the obtained sequence encodes a conserved MYB-type protein, providing a foundation for subsequent phylogenetic comparison, subcellular localization, and functional analyses.

### 3.2 Alignment of *PpMYB4* amino acid sequences and phylogenetic tree analysis

**Figure 1:**
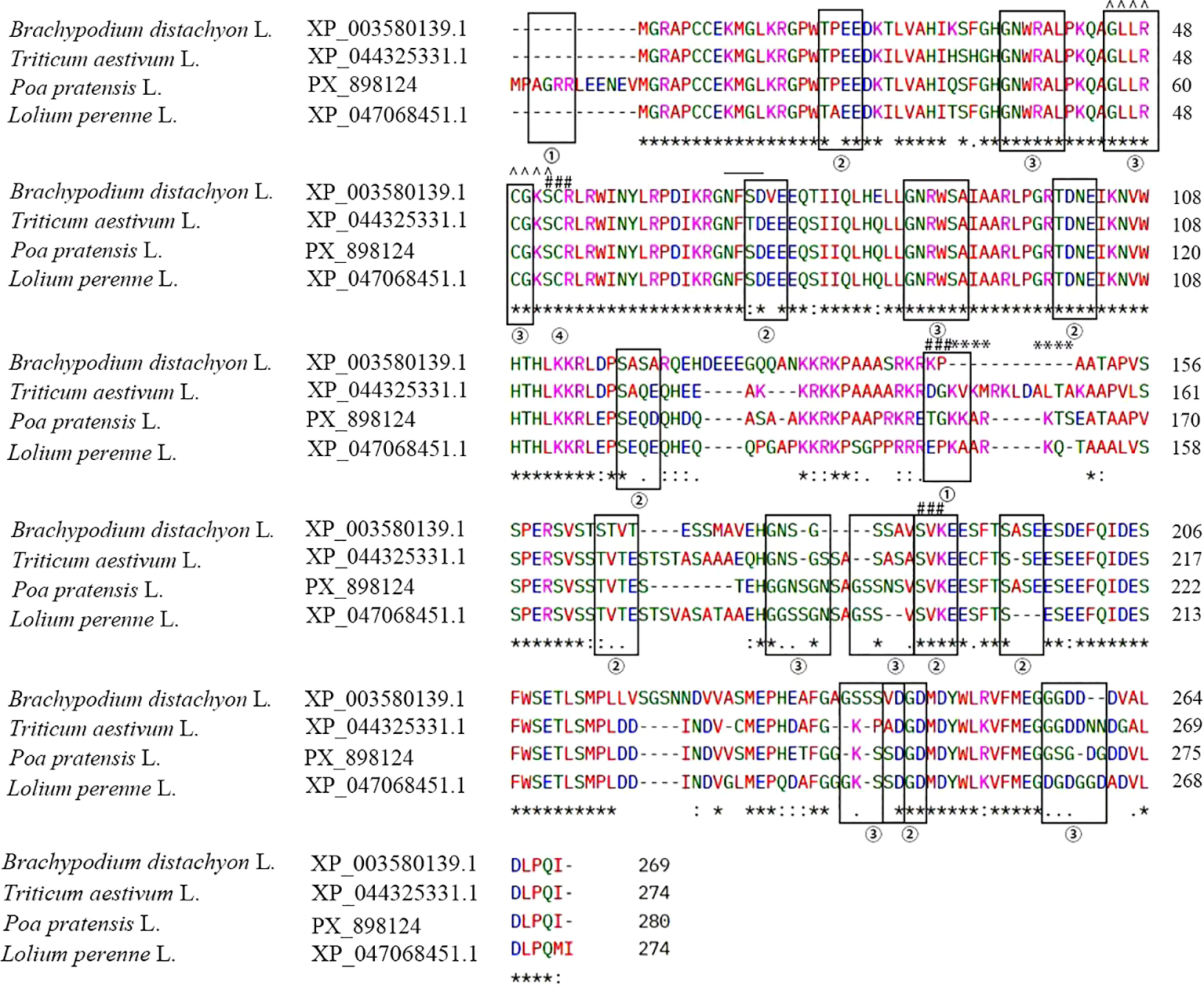
Multiple sequence alignment of MYB4 amino acid sequences from *P. pratensis* (PX_898124), *B. distachyon* (XP_003580139.1), *T. aestivum* (XP_044325331.1), and *Lolium perenne* (XP_047068451.1). Functional modification sites are annotated: ① indicates amidation site; ② indicates casein kinase II phosphorylation site; ③ indicates N-myristoylation site; # indicates protein kinase C phosphorylation site; * indicates cAMP/cGMP-dependent protein kinase phosphorylation site; ^ indicates protein kinase ATP-binding region.

To investigate the evolutionary conservation of *PpMYB4* and clarify its phylogenetic relationship within *Poaceae*, *PpMYB4* was compared with representative MYB4 homologs from multiple grass species. Multiple sequence alignment showed that *PpMYB4* contains several highly conserved regions (**Figure 1**), particularly within the N-terminal MYB DNA-binding domain, consistent with typical R2R3-MYB transcription factors. In contrast, the C-terminal region exhibited higher sequence variability, suggesting potential divergence in regulatory motifs and functional specificity among species.

**Figure 2:**
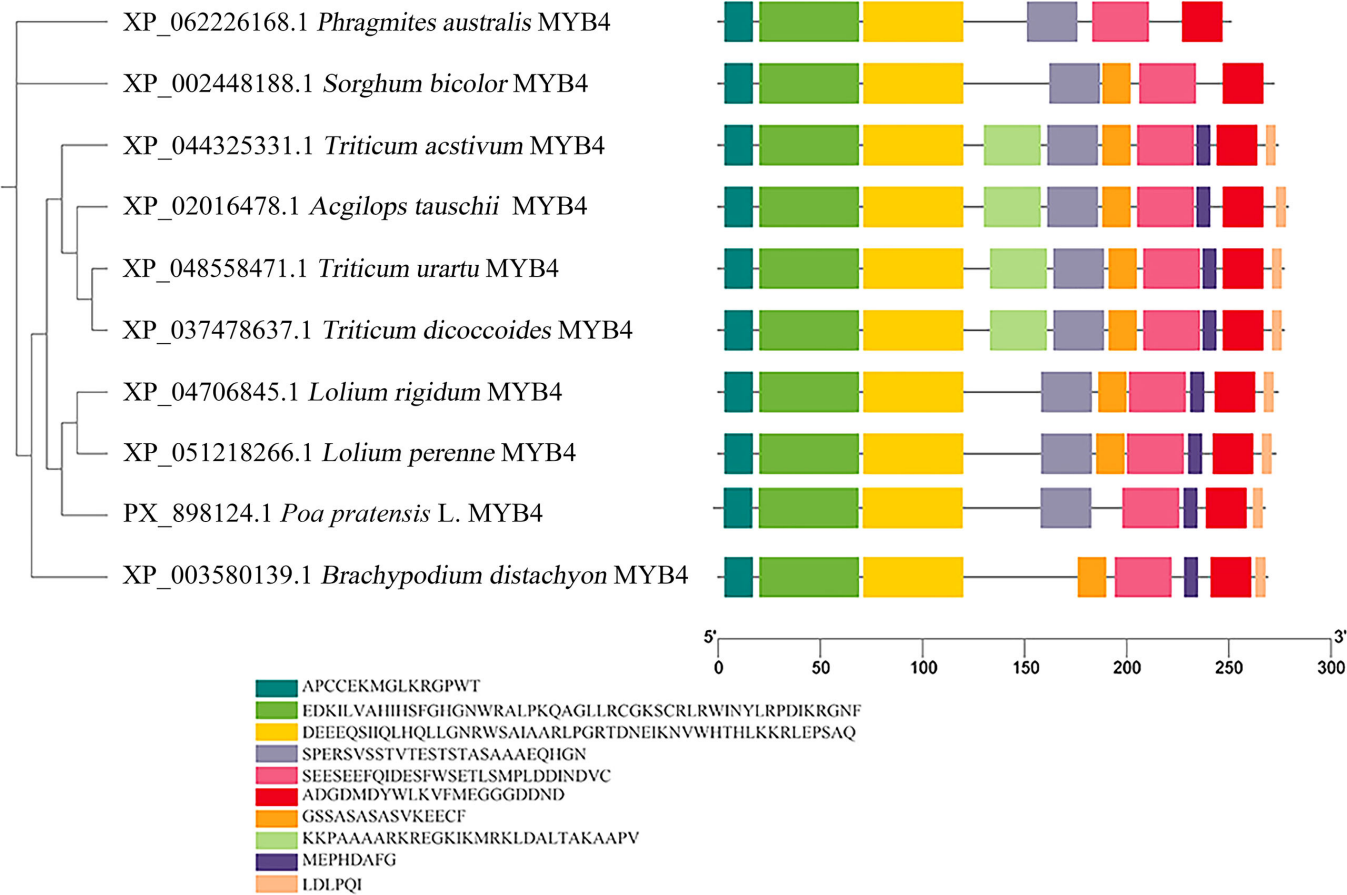
Phylogenetic analysis of MYB4 proteins from Kentucky bluegrass and multiple species.

A phylogenetic tree was subsequently constructed based on full-length protein sequences to infer relatedness. The resulting topology indicated that *PpMYB4* (PX898124) clusters closely with BdMYB4 from *Brachypodium distachyon* (XP_003580139.1), LrMYB4 from *Lolium rigidum* (XP_047068451.1), and TaMYB4 from *Triticum aestivum* (XP_044325331.1) (**Figure 2**), supporting that *PpMYB4* is a conserved MYB4 homolog in Poaceae. Together, these analyses provide evidence for strong sequence conservation of *PpMYB4* in the canonical MYB domain and establish its close evolutionary proximity to MYB4 proteins from related grass species, thereby supporting subsequent functional characterization.

### 3.3 Subcellular localization of *PpMYB4*

To determine the subcellular localization of *PpMYB4*, the empty vector pCAMBIA1300-EGFP and the fusion construct pCAMBIA1300-*PpMYB4*-EGFP were transiently expressed in *N. benthamiana* leaves via *A. tumefaciens*-mediated infiltration. Fluorescence signals were examined by confocal laser scanning microscopy.

**Figure 3:**
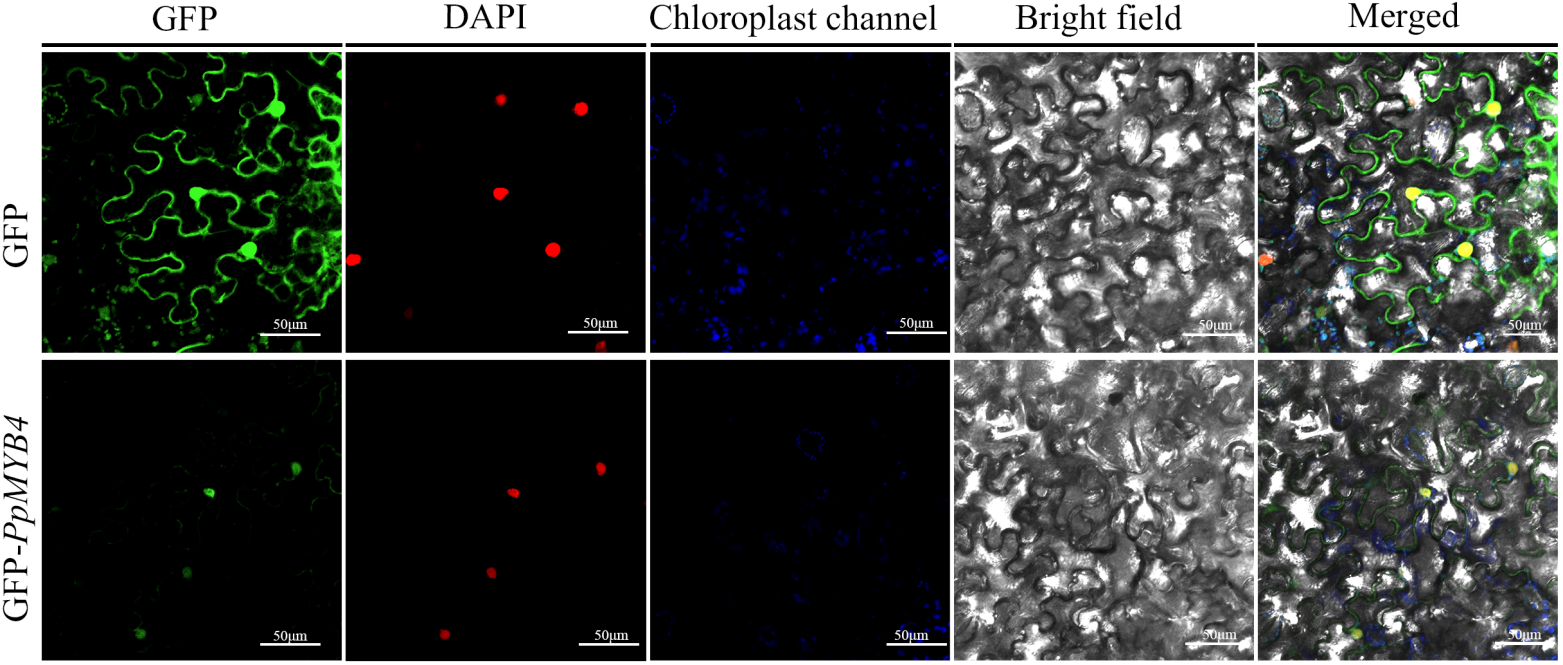
Subcellular localization of *PpMYB4* in *N. benthamiana* leaf epidermal cells. Upper panels: cells expressing the empty vector control pCAMBIA1300-EGFP. Lower panels: cells expressing the pCAMBIA1300-*PpMYB4*-EGFP fusion construct. Columns from left to right: GFP fluorescence (488 nm excitation), DAPI fluorescence (405 nm excitation, nuclear marker), chloroplast autofluorescence (633 nm excitation), bright field, and merged overlay. Scale bar = 50 µm.

In control cells expressing EGFP alone, green fluorescence was broadly distributed throughout the cytoplasm and nucleus, consistent with the expected diffuse distribution of free GFP (**Figure 3**, upper panels). In contrast, the *PpMYB4*-EGFP fusion signal was predominantly concentrated in a discrete intracellular compartment (**Figure 3**, lower panels). To unequivocally identify this compartment as the nucleus, DAPI co-staining was performed. The *PpMYB4*-EGFP fluorescence signal precisely overlapped with the DAPI-stained nuclear signal (**Figure 3**, merged panels), confirming that *PpMYB4* is localized to the nucleus. This localization pattern is consistent with the predicted function of *PpMYB4* as a transcription factor and aligns with reports of nuclear targeting for MYB4 proteins in canola (Chen et al., 2016) and petunia (Colquhoun et al., 2011).

### 3.4 Expression pattern analysis of the *PpMYB4* gene in Kentucky bluegrass

**Figure 4:**
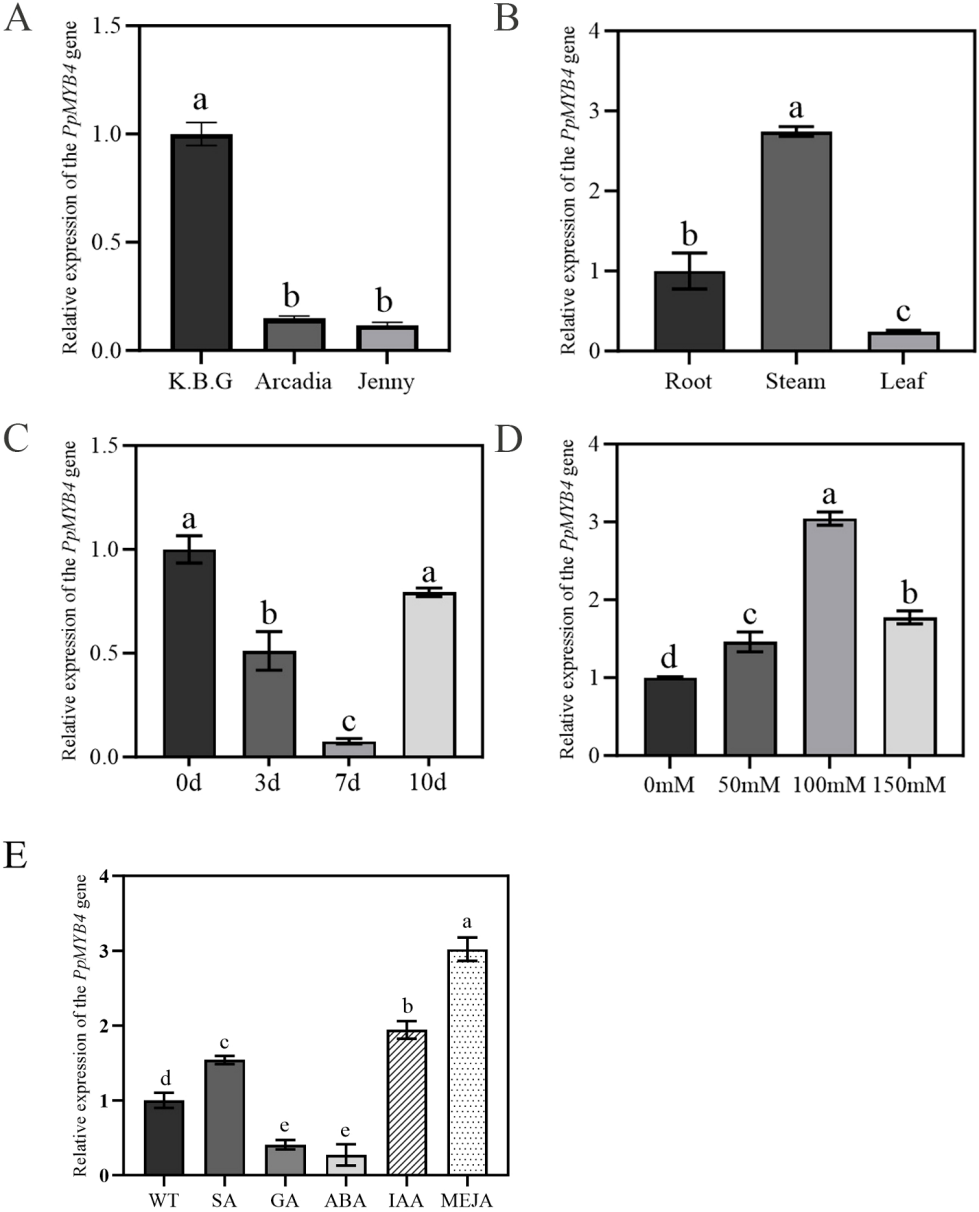
Expression pattern analysis of *PpMYB4*. **(A)** Differential expression of *PpMYB4* among three cultivars (‘K.B.G’, ‘Arcadia’, ‘Jenny’). **(B)** Tissue-specific expression in root, stem, and leaf of ‘K.B.G’. **(C)** Expression under progressive drought stress (0, 3, 7, 10 d of natural dehydration). **(D)** Expression under saline-alkali stress (0, 50, 100, 150 mM, 3 d treatment). **(E)** Expression following exogenous hormone treatments (untreated control, SA 200 mg/L, GA 200 mg/L, ABA 30 mg/L, IAA 800 mg/L, MeJA 50 mg/L; 5 d foliar spray). Data represent mean ± SE (n = 3 biological replicates, each with three technical replicates). Different lowercase letters indicate statistically significant differences (*P* < 0.05, one-way ANOVA, Duncan’s multiple range test).

To characterize the transcriptional features of *PpMYB4* in Kentucky bluegrass, its expression was quantified by RT–qPCR across cultivars, tissues, and under abiotic stress and phytohormone treatments. As shown in **Figure 4A**, *PpMYB4* transcript abundance differed significantly among the three tested cultivars (*P<* 0.05), with the expression ranking ‘K.B.G’ > ‘Arcadia’ > ‘Jenny’. Notably, *PpMYB4* expression in ‘K.B.G’ was 6.73-fold and 8.67-fold higher than that in ‘Arcadia’ and ‘Jenny’, respectively, indicating strong genotype-dependent expression. Given its consistently highest expression, ‘K.B.G’ was selected for subsequent tissue- and stress-related assays.

Tissue-specific expression analysis revealed that *PpMYB4* was detectable in roots, stems, and leaves, but at markedly different levels (**Figure 4B**). The highest expression was observed in stems, followed by roots, with the lowest level in leaves (stem > root > leaf; *P* < 0.05). Stem expression was approximately 11.3-fold higher than leaf expression, suggesting pronounced tissue preference and implying a potential role for *PpMYB4* in stem-associated developmental or physiological processes.

To evaluate whether *PpMYB4* participates in abiotic stress responses, plants were exposed to drought and saline–alkali stress. Under drought treatment, *PpMYB4* expression showed a dynamic pattern, with an initial decrease followed by a subsequent increase over time (**Figure 4C**; *P* < 0.05), indicating that *PpMYB4* may be regulated in a stage-dependent manner during progressive water deficit. Under saline–alkali conditions, *PpMYB4* transcripts were significantly induced relative to the untreated control (**Figure 4D**; *P* < 0.05). Expression increased under all tested concentrations (50, 100, and 150 mM), with the highest induction at 100 mM, supporting that *PpMYB4* is responsive to saline–alkali stress.

Because promoter analysis predicted the presence of multiple hormone-responsive cis-elements, *PpMYB4* expression was further examined following exogenous hormone application (**Figure 4E**). RT-qPCR results showed that SA, IAA, and MeJA treatments significantly elevated *PpMYB4* transcript levels to 1.54 ± 0.12-fold, 1.94 ± 0.21-fold, and 3.02 ± 0.38-fold, respectively, compared with the untreated control (0 d) (*P<* 0.05, one-way ANOVA followed by Duncan’s multiple range test). Among the tested hormones, MeJA elicited the strongest induction, suggesting that the JA signaling pathway may be a primary upstream regulator of *PpMYB4* transcription. In contrast, GA and ABA treatments significantly suppressed *PpMYB4* expression to 0.41 ± 0.07-fold and 0.27 ± 0.05-fold of the baseline, respectively (*P* < 0.05). Collectively, these results indicate that *PpMYB4* is positively regulated by defense-associated hormones (SA, MeJA) and growth-promoting auxin (IAA) while being repressed by ABA and GA, suggesting a role at the intersection of growth–defense hormonal crosstalk in Kentucky bluegrass.

### 3.5 Phenotypic characterization and stress-related traits of *PpMYB4*-overexpressing Kentucky bluegrass

**Figure 5:**
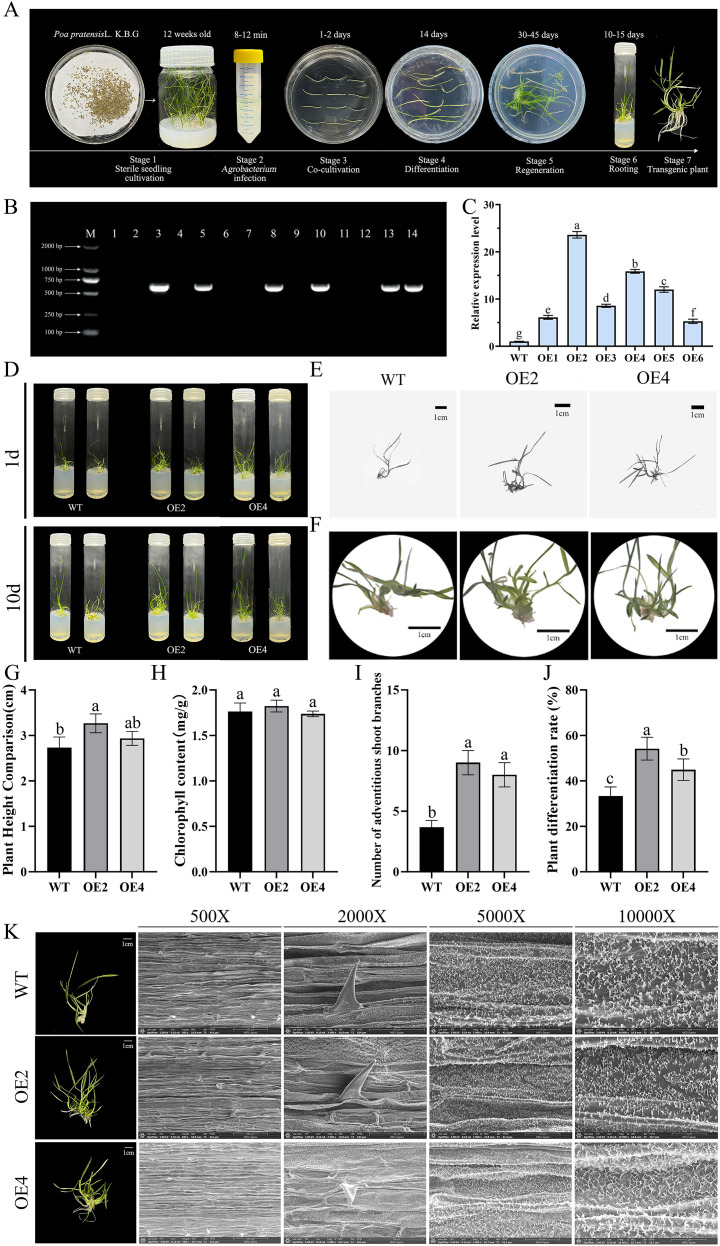
Growth and development analysis of transgenic Kentucky bluegrass. **(A)** Stages of *A. tumefaciens* -mediated transformation. **(B)** DNA molecular detection of overexpression lines. **(C)** Expression analysis of *PpMYB4* in overexpressed transgenic Kentucky bluegrass. **(D)** Plants harvested at 50 and 60 days after co-cultivation. **(E)** Morphological structure after 60 days of co-culture. **(F)** Clustered shoot structure after 60 days of co-culture. **(G)** Mean plant height after 60 days of co-culture. **(H)** Chlorophyll content after 60 days of co-culture. **(I)** Mean branch number per clustered shoot after 60 days of co-culture. **(J)** Mean differentiation rate after 60 days of co-culture. **(K)** Leaf ultrastructural analysis after 60 days of co-culture. Data represent mean ± SE (n = 3 biological replicates, each with three technical replicates). Different lowercase letters indicate statistically significant differences (*P* < 0.05, Student’s *t*-test or one-way ANOVA followed by Duncan’s multiple range test, as appropriate).

Molecular validation of transgenic lines. Among the hygromycin-resistant regenerated plants, PCR amplification using *hpt*-specific primers confirmed transgene insertion in multiple independent lines (**Figure 5B**). Six independent lines (OE1-OE6) were selected for further analysis. Under non-stress conditions, qRT-PCR revealed that mean expression levels were significantly higher than those in the wild type (WT), with OE2 and OE4 exhibiting the highest expression levels, which were 23.6 and 15.86 times that of WT, respectively (*P<* 0.01, Student’s t-test), confirming successful overexpression (**Figure 5C**). Semi-quantitative RT-PCR further corroborated the elevated *PpMYB4* transcript abundance in all twe OE lines relative to WT (**Supplementary File 1**).

Phenotypic analysis under normal growth conditions. Transgenic Kentucky bluegrass plants overexpressing *PpMYB4* were evaluated after 60 d of co-cultivation (**Figure 5A**). Under identical growth conditions, *PpMYB4* overexpression did not cause obvious morphological abnormalities compared with WT plants. The mean plant height of OE lines (6.18 ± 0.42 cm) was significantly higher than that of WT (4.78 ± 0.35 cm) (*P* < 0.05; **Figures 5E, G**), although overall plant architecture remained comparable between genotypes.

Root-related performance was improved in OE lines: the rooting rate reached 68.75% in OE plants versus 53.85% in WT (**Figure 5E**), indicating that PpMYB4 may promote root initiation during regeneration. By contrast, the differentiation rate was reduced in WT plants relative to OE (**Figure 5J**), suggesting that the expression of the *PpMYB4* gene may promote the differentiation process within this regeneration system. Chlorophyll content showed no significant difference between OE and WT plants (*P* = 0.32, Student’s t-test; **Figure 5H**), implying that *PpMYB4* overexpression does not markedly affect chlorophyll accumulation under non-stress conditions.

Microscopic observation revealed enhanced tillering/branching potential in OE plants: the average number of branches per clustered shoot in OE plants was 10.33 ± 1.15, which was 1.51-fold higher than that of WT (6.83 ± 0.76) (*P* < 0.05; **Figures 5F, I**). Consistently, the growth rate calculated between 50 and 60 d post co-cultivation was higher in OE plants (0.31 ± 0.03 cm/d) than in WT (0.21 ± 0.02 cm/d) (*P* < 0.05; **Figure 5D**), supporting a positive role of *PpMYB4* in vegetative growth.

Leaf epidermal wax accumulation. To explore potential stress-related structural adaptations, leaf epidermal ultrastructure was examined by SEM. OE plants exhibited noticeably greater epicuticular wax abundance on the leaf surface than WT (**Figure 5K**). Quantitative analysis of papilla size was conducted using ImageJ. Specifically, the ImageJ software was utilized to measure the papilla dimensions (length, width, height) from scanning electron microscopy (SEM) images at a magnification of 5000 times (n = 30 papillae from three biological replicates for each genotype). Assuming an elliptical geometric shape to estimate the volume, the calculation formula is: $V=\frac{4}{3}\pi \cdot \frac{l}{2}\cdot \frac{w}{2}\cdot \frac{h}{2}$. The results indicate that the estimated papilla volume in the OE plants is 1.13 ± 0.08 times that of the WT (*P* = 0.038, Student’s t-test). Given the established role of cuticular wax in reducing non-stomatal water loss (Gong et al., 2017; Lee and Suh, 2015), these observations suggest that *PpMYB4* overexpression may enhance drought tolerance, at least in part, by promoting wax accumulation on the leaf epidermis.

**Figure 6:**
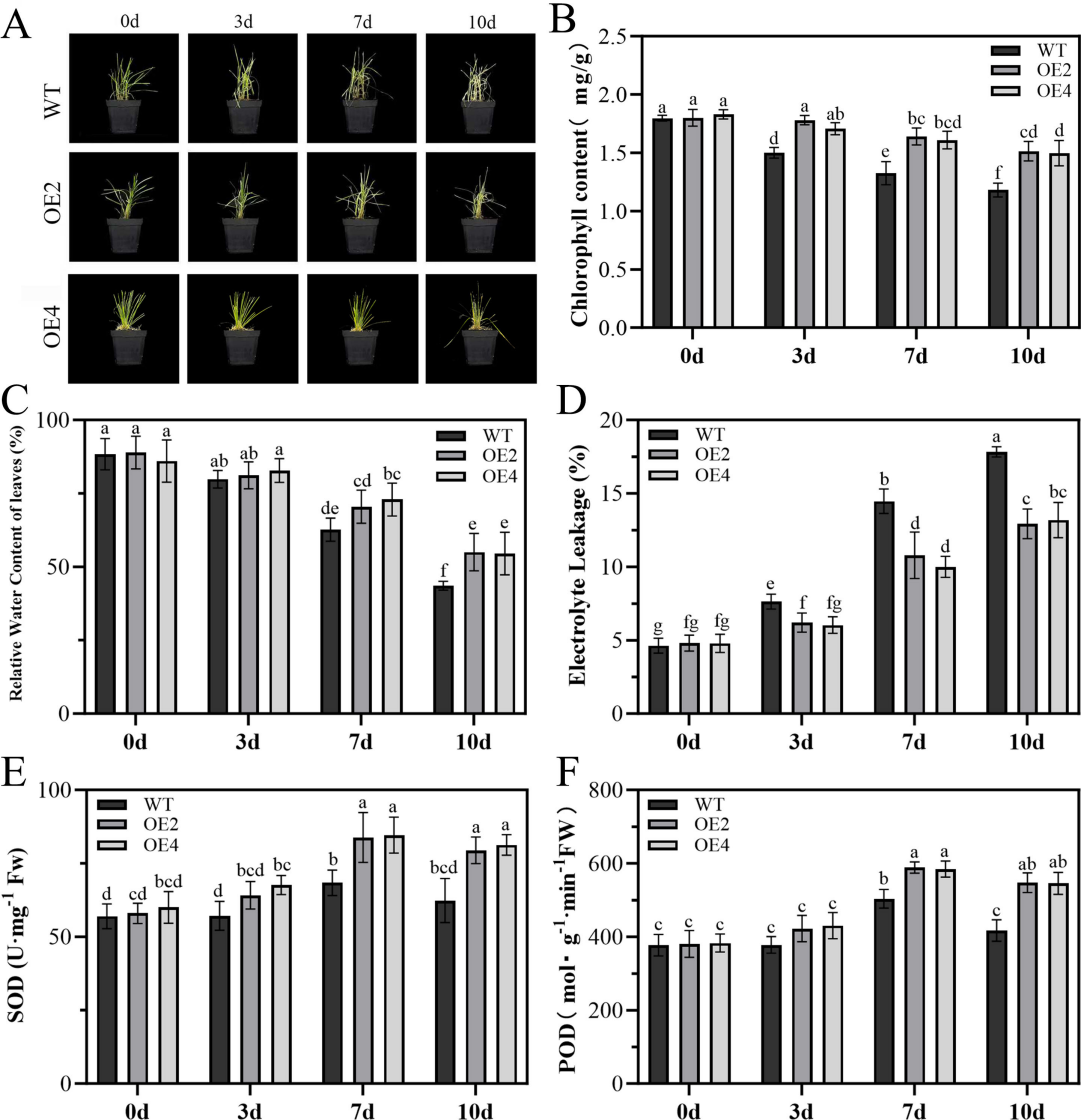
Analysis of stress resistance in *PpMYB4*-overexpressing Kentucky bluegrass under drought stress (10% PEG 6000). **(A)** Representative growth phenotypes of wild-type (WT) and *PpMYB4*-overexpressing (OE) plants at 0, 3, 7, and 10 d after stress initiation. **(B)** Chlorophyll content (mg/g). **(C)** Leaf relative water content (%). **(D)** Electrolyte leakage (%). **(E)** Superoxide dismutase (SOD) activity (U·g^-1^ FW). **(F)** Peroxidase (POD) activity (µmol·g^-1^·min^-1^ FW). Data represent mean ± SE (n = 3 biological replicates). Different lowercase letters indicate statistically significant differences among time points within each genotype, and asterisks indicate significant differences between WT and OE at the same time point (*P* < 0.05, Student’s *t*-test or one-way ANOVA followed by Duncan’s multiple range test, as appropriate).

Drought stress response of *PpMYB4*-overexpressing lines. Under drought stress simulated by 10% PEG 6000 treatment, OE lines exhibited significantly better drought-resistant phenotypes compared with WT (**Figure 6A**). After 10 d of stress, WT plants showed obvious wilting and yellowing, whereas OE lines maintained relatively vigorous growth. Physiological measurements further elucidated the drought-tolerance mechanisms mediated by *PpMYB4* (**Figures 6B–F**). The results of chlorophyll content measurement indicate that, with the extension of stress duration, the chlorophyll content in the WT is significantly lower than that in the OE (**Figure 6B**; *P* < 0.05).Throughout the stress period, leaf relative water content (RWC) in OE lines was significantly higher than in WT at each time point (**Figure 6C**; *P* < 0.05), indicating enhanced water retention capacity. Concurrently, electrolyte leakage, a widely used indicator of membrane integrity, was significantly lower in OE lines than in WT (**Figure 6D**; *P* < 0.05), demonstrating that *PpMYB4* effectively maintains cell membrane stability under dehydration. With respect to antioxidant enzyme activities, superoxide dismutase (SOD) activity in OE lines was significantly higher than in WT at 7 d and 10 d of stress (**Figure 6E**; *P* < 0.05), suggesting an enhanced capacity for superoxide radical scavenging. Similarly, peroxidase (POD) activity was elevated in OE lines at later stress stages (7 d and 10 d; **Figure 6F**; *P* < 0.05), supporting enhanced detoxification of hydrogen peroxide. Collectively, these physiological data indicate that *PpMYB4* overexpression simultaneously improves water retention, preserves membrane integrity, and reinforces enzymatic ROS-scavenging capacity, thereby conferring robust drought tolerance in Kentucky bluegrass.

### 3.6 Cloning and bioinformatics analysis of *PpMYB4* promoter

To isolate the upstream regulatory region of *PpMYB4*, promoter-specific primers were designed based on the Kentucky bluegrass reference genome. PCR amplification generated a single fragment of 1766 bp, which was gel-purified, inserted into the pBI121 vector, and confirmed by Sanger sequencing (**Supplementary File 1**), indicating successful acquisition of the *PpMYB4* promoter sequence.

**Figure 7:**
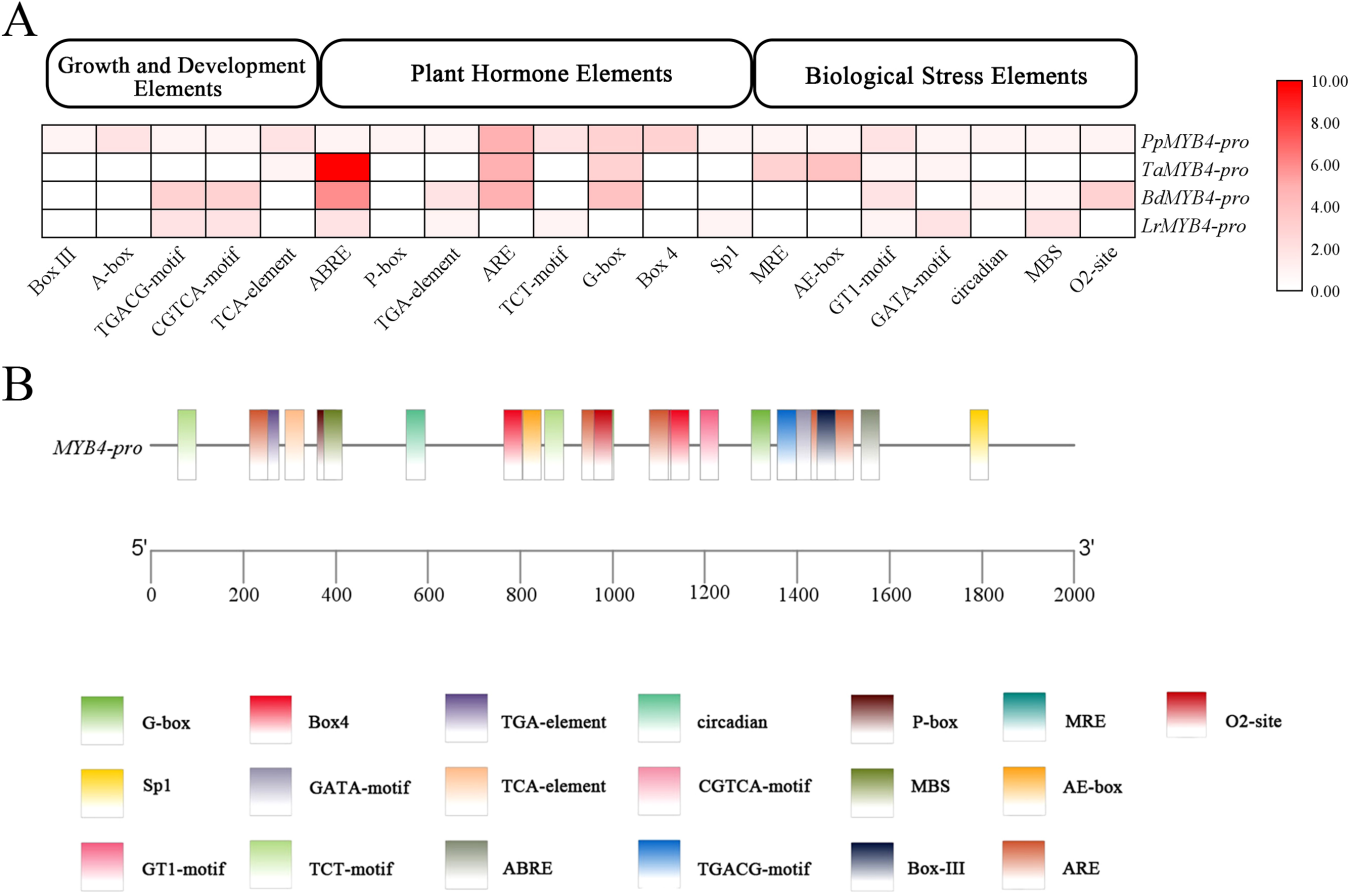
Cis-acting element analysis in the *PpMYB4* promoter region. **(A)** Numbers of cis-acting elements in the promoter region. **(B)** Distribution and predicted functions of cis-acting elements in the promoter region. Note: Pp, *P. pratensis* L.; Ta, *Triticum aestivum*; Bd, *Brachypodium distachyon*; Lr, *Lolium rigidum*.

Cis-acting element prediction using PlantCARE revealed that the *PpMYB4* promoter harbors diverse regulatory motifs associated with plant development, hormone signaling, light responsiveness, and abiotic stress responses (**Figure 7**). Multiple hormone-related elements were identified, including TGACG-motif and CGTCA-motif (MeJA responsiveness), TCA-element (SA responsiveness), ABRE (ABA responsiveness), P-box (GA responsiveness), and TGA-element (auxin responsiveness), suggesting that *PpMYB4* transcription may be modulated by several phytohormone pathways. In addition, abundant light-responsive elements (e.g., G-box, Box 4, Sp1, MRE, AE-box, GT1-motif, and GATA-motif) were detected, implying potential regulation by light cues. The promoter also contained stress-associated elements such as ARE (anaerobic induction) and MBS (MYB-binding site involved in drought inducibility), together with circadian and O2-site motifs related to rhythmic regulation and metabolism/development. Collectively, these in silico analyses support that the *PpMYB4* promoter integrates multiple environmental and hormonal signals and may underlie the observed inducible expression of *PpMYB4* under drought and saline–alkali stress conditions.

### 3.7 Identification of the core promoter region of *PpMYB4* and its drought-inducible activity

**Figure 8:**
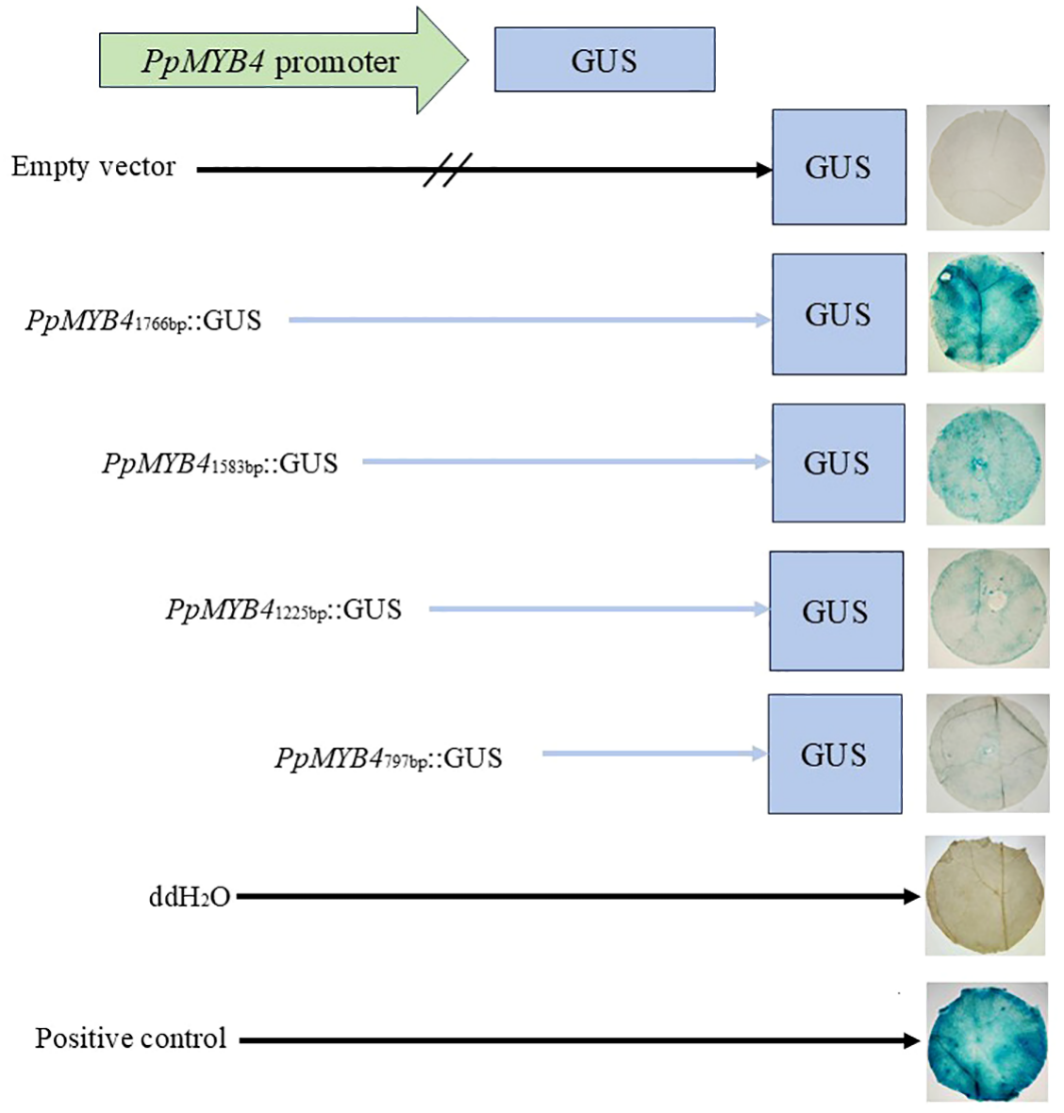
Activity verification of the full-length PpMYB4 promoter and its 5’-end deletion fragments.

To define the core regulatory region responsible for *PpMYB4* transcription, the promoter sequence was analyzed using the BDGP program, which predicted five putative core promoter regions with high confidence scores (**Table 1**). To experimentally validate promoter activity and delimit functional segments, the full-length *PpMYB4* promoter (GUS–*MYB4*, 1766 bp) and three 5′-deletion derivatives were constructed (**Figure 8A**, schematic): F1/R (-1200 to +1, retaining MBS, ABRE, and partial TGACG-motif elements), F2/R (-700 to +1, retaining ABRE and partial ARE elements), and F3/R (-300 to +1, retaining only the minimal TATA-box region). Each fragment was fused upstream of the GUS reporter gene in the pBI121 vector and transiently expressed in *N. benthamiana* leaves.

**Table 1:** Prediction of core promoter regions.

Start	End	Score	Promoter sequence
57	107	0.81	TATTGGTGTTTTACAAGGTGCGGGACCGACTGGAACGTTG(A)ACGCGAGTT
604	654	0.83	ATTTACTATAAATAAAATTTCCCTTAAAAATTTCCAGCAC(A)ATCTTCTAA
618	668	0.97	AAATTTCCCTTAAAAATTTCCAGCACAATCTTCTAACCCC(T)CGCATGAAA
935	985	0.95	TACGGGAAGATATATGAGTCGTACCCACACCGTGAGAAAC(C)CTCAAACCC
1549	1599	0.99	GCGGCAGGCATAAATACCTGGACGCCCGGCGACTCCGGC(A)TTACTCTGC

Histochemical GUS staining revealed that the untreated control (CK) and the empty vector (pBI121) control showed only trace background staining with no detectable difference between them (**Figure 8**). The full-length promoter drove strong GUS staining, confirming robust transcriptional activity in planta. Compared with the full-length promoter, the F1/R deletion exhibited a pronounced reduction in staining intensity, and GUS signals in the F2/R and F3/R constructs were further weakened to near-background levels (**Figure 8**). This progressive attenuation of reporter activity with increasing 5’ truncation indicates that cis-regulatory elements critical for high-level transcription are enriched in the -1766 to -1200 bp region and that this upstream segment contains the major functional core/activation region sustaining promoter strength.

## 4 Discussion

The MYB gene family constitutes one of the largest groups of transcription factors in plants and is widely implicated in developmental regulation and environmental adaptation (Aadel et al., 2021). In this study, we cloned an R2R3-MYB transcription factor gene, *PpMYB4*, from Kentucky bluegrass and performed integrated analyses including subcellular localization, expression profiling under abiotic stresses and hormone treatments, phenotypic characterization of *PpMYB4*-overexpressing plants, and functional dissection of the *PpMYB4* promoter. Collectively, our results support that *PpMYB4* is a nucleus-localized transcription factor that is transcriptionally regulated by drought/saline–alkali stress and multiple phytohormones, and that its overexpression is associated with enhanced vegetative growth traits and increased epicuticular wax deposition, suggesting a potential role in drought adaptation.

### 4.1 Nuclear localization and evolutionary conservation support a transcriptional regulatory role of *PpMYB4*

Subcellular localization analysis showed that the *PpMYB4*–EGFP fusion protein was predominantly detected in the nucleus, consistent with its presumed function as a transcription factor and with previous reports of MYB4 proteins exhibiting nuclear localization in other species (Colquhoun et al., 2011; Chen et al., 2016). In addition, sequence alignment and phylogenetic analysis indicated that *PpMYB4* is closely related to *MYB4* homologs from other Poaceae members (e.g., *Brachypodium*, wheat, and *Lolium*), suggesting that *PpMYB4* may share conserved regulatory functions in grasses (Sukumaran et al., 2023; Wani et al., 2025). Notably, the conservation in the N-terminal *MYB* DNA-binding region, together with divergence in the C-terminal region, is consistent with a model in which DNA-binding specificity is largely conserved while transcriptional regulatory potential and protein–protein interaction capacity may vary among species, potentially contributing to functional diversification (Wu et al., 2022; Mitra et al., 2021).

### 4.2 Tissue-preferential expression suggests context-specific roles in vegetative organs

Many *MYB4* genes display tissue-biased expression patterns, reflecting functional partitioning across organs (Yu et al., 2021; Li et al., 2024). Here, *PpMYB4* transcripts were detectable in roots, stems, and leaves, with the highest accumulation in stems. This stem-preferential expression resembles reports in some cereal species and suggests that *PpMYB4* may be associated with stem-related physiological processes such as vascular development, secondary metabolism, or structural reinforcement (Ma et al., 2025; Lee et al., 2025; Zhao et al., 2024; Yu et al., 2021). The relatively low expression in leaves under normal conditions does not exclude an important leaf function under stress, as stress-induced transcriptional activation may dominate functional output in aerial tissues (Liu et al., 2015).

### 4.3 *PpMYB4* integrates hormonal and abiotic stress signals at the transcriptional level

RT-qPCR analysis revealed that *PpMYB4* is responsive to both drought and saline–alkali stress, positioning it within abiotic stress signaling networks in Kentucky bluegrass. The dynamic expression pattern under drought-initial decrease at 3 d followed by significant upregulation at 7 and 10 d-implies phase-dependent regulation. The early suppression may reflect resource reallocation from growth to immediate osmotic adjustment, while the subsequent upregulation likely represents activation of protective transcriptional programs, including wax biosynthesis and antioxidant defense, during prolonged water deficit (Tang et al., 2025; Zhang et al., 2023a). Under saline–alkali stress, *PpMYB4* was induced across tested concentrations, consistent with its involvement in osmotic/ionic stress responses (Zhang et al., 2024b).

Hormone treatments further revealed that *PpMYB4* is differentially regulated by multiple signaling pathways: SA, IAA, and MeJA promoted its expression, whereas ABA and GA repressed it. The strongest induction by MeJA (3.02-fold) is consistent with the presence of TGACG-motif and CGTCA-motif (MeJA-responsive elements) in the *PpMYB4* promoter and with the established role of JA signaling in activating MYB-mediated secondary metabolism and cuticular wax biosynthesis (Wang et al., 2024; Song et al., 2022). In *Arabidopsis*, JA-responsive MYB transcription factors (e.g., *MYB94*, *MYB96*) directly activate wax biosynthetic genes such as *CER1* and *KCS* (Lee and Suh, 2015). The strong MeJA responsiveness of *PpMYB4* thus suggests that it may function downstream of JA signaling to promote wax deposition as a defense-related drought adaptation.

IAA-mediated induction (1.94-fold) is consistent with the TGA-element (auxin-responsive) identified in the *PpMYB4* promoter. Auxin is well known to promote root development and cell expansion (Overvoorde et al., 2010), which aligns with the enhanced rooting rate observed in OE lines. This suggests that *PpMYB4* may integrate auxin signals to coordinate root growth with stress preparedness.

SA-mediated induction (1.54-fold) may reflect a node of biotic–abiotic stress crosstalk. SA has been shown to prime antioxidant defenses and enhance tolerance to secondary abiotic stresses (Sharma et al., 2020). Given that OE lines exhibited elevated SOD and POD activities under drought, the SA-*PpMYB4* axis may contribute to antioxidant enzyme priming.

The ABA-mediated repression (0.27-fold) is particularly noteworthy because ABA is canonically regarded as the primary drought-response hormone (Yang et al., 2012). This apparent paradox may be explained in several ways: (i) *PpMYB4* may operate primarily through ABA-independent drought tolerance pathways, analogous to the DREB2/CBF pathway in cereals; (ii) ABA may exert negative feedback on *PpMYB4* to prevent excessive wax accumulation that could impair stomatal gas exchange; or (iii) the antagonistic relationship between ABA and MYB4 may reflect the ABA-MYB4 module described in kiwifruit, where ABA antagonizes MYB4-mediated transcriptional repression to activate suberization-related genes (Wei et al., 2021). Future experiments employing ABA biosynthesis inhibitors (e.g., fluridone), ABA signaling mutants, and targeted mutagenesis of the ABRE elements in the *PpMYB4* promoter will be required to distinguish among these possibilities (Li et al., 2012, Li et al., 2021).

### 4.4 Overexpression phenotypes imply links to growth vigor and epidermal protection

Constitutive expression of *PpMYB4* did not cause obvious developmental abnormalities but was associated with improved vegetative growth (higher growth rate, increased branching, and enhanced rooting) and two key drought-adaptive traits: increased epicuticular wax deposition and elevated antioxidant enzyme activities.

Wax-mediated structural protection. SEM analysis revealed that OE lines accumulated more epicuticular wax on leaf surfaces than WT. Cuticular wax is a well-established barrier against non-stomatal water loss, and its role in drought tolerance has been documented in multiple species, including rice (*OsMYB60*) (Jian et al., 2022), *Arabidopsis* (*MYB94*, *MYB96*) (Lee and Suh, 2015), wheat (*TaMYB60*) (Wang et al., 2024), and poplar (*PtoMYB142*) (Song et al., 2022). In Kentucky bluegrass, *PpCER1* has been identified as a key wax alkane biosynthetic gene contributing to drought tolerance (Wang et al., 2021a). We hypothesize that *PpMYB4* may transcriptionally activate *PpCER1* or other wax-pathway genes (e.g., *KCS*, *CER3*, *MAH1*) to enhance wax production, although direct target identification via chromatin immunoprecipitation (ChIP) or electrophoretic mobility shift assay (EMSA) is needed to confirm this regulatory relationship.

Antioxidant enzyme-mediated biochemical protection. The significantly elevated SOD and POD activities in OE lines at 7 and 10 d of drought stress (**Figures 6E, F**) indicate that *PpMYB4* enhances the enzymatic ROS-scavenging capacity. Under drought, excessive ROS accumulation causes lipid peroxidation, protein denaturation, and DNA damage, leading to membrane destabilization and cell death (Devireddy et al., 2021). The lower electrolyte leakage observed in OE lines (**Figure 6D**) corroborates that *PpMYB4*-mediated antioxidant enhancement effectively preserves membrane integrity. This observation is consistent with the role of *MYB4* in activating antioxidant-related genes. For example, *Arabidopsis MYB4* was shown to upregulate chelating peptide synthesis genes (*PCS1*, *MT1C*) to synergistically enhance antioxidant defense under cadmium stress (Agarwal et al., 2020). In rice, ectopic expression of *OsMYB4* enhanced proline accumulation and antioxidant enzyme activities in transgenic *Arabidopsis* (Lian et al., 2021). Similarly, in high-altitude Himalayan rice, *MYB4* upregulation was coupled with time-dependent ROS regulation under cold stress (Wani et al., 2025).

We propose that *PpMYB4* confers drought tolerance through a dual mechanism: (i) structural protection via enhanced wax deposition that reduces water loss from the leaf surface, and (ii) biochemical protection via elevated antioxidant enzyme activities that mitigate oxidative damage. These two mechanisms are complementary and may be coordinately regulated—for instance, wax accumulation slows the rate of dehydration, providing a longer temporal window during which antioxidant defenses can be mobilized. The hormone-responsive expression profile of *PpMYB4* (particularly JA induction and ABA repression) suggests that it may function at a hormonal signaling node that balances growth with dual-layered stress protection. Future transcriptomic analysis (RNA-seq) of OE versus WT lines under drought conditions would help identify the complete set of *PpMYB4* target genes and clarify the relative contributions of wax and antioxidant pathways. It should be noted that protein-level confirmation of *PpMYB4* overexpression (e.g., by Western blot) was not performed due to the current lack of a validated anti-*PpMYB4* antibody. Although the qRT-PCR and semi-quantitative RT-PCR data provide strong evidence for transcriptional overexpression, future studies should develop custom antibodies or employ epitope-tagged constructs to verify PpMYB4 protein accumulation.

### 4.5 The *PpMYB4* promoter contains an upstream region critical for high activity and is drought inducible

Promoter cloning and transient expression assays using 5’-deletion fragments demonstrated that promoter activity decreased progressively as the 5’ region was truncated, indicating that key cis-regulatory elements required for strong transcription are enriched in the upstream segment (Banerjee et al., 2015; Leong et al., 2013). Moreover, PEG-induced osmotic stress increased promoter-driven GUS activity and GUS transcript abundance, supporting that the *PpMYB4* promoter is drought inducible (Zhang et al., 2016). Together with the presence of drought-related motifs (e.g., MBS), these results suggest that drought responsiveness may be mediated by MYB-related transcriptional modules and that *PpMYB4* itself could participate in regulatory feedback or feedforward loops within stress networks.

## 5 Conclusion

In summary, our results indicate that *PpMYB4* is a Poaceae-conserved, nucleus-localized R2R3-MYB whose transcription is regulated by abiotic stresses and multiple hormones. Overexpression of *PpMYB4* is associated with enhanced growth traits and increased leaf epicuticular wax deposition, and the *PpMYB4* promoter contains an upstream region essential for strong activity and is inducible by drought-related osmotic stress. These findings provide a foundation for dissecting the *PpMYB4*-centered regulatory network and for developing molecular strategies to improve drought resilience in Kentucky bluegrass.

## Data Availability

The original contributions presented in the study are included in the article/**Supplementary Material**, further inquiries can be directed to the corresponding author/s.
