# Supplementary material for: PpMYB4-mediated regulation of wax accumulation and abiotic stress responses in Kentucky bluegrass (Poa pratensis L.)
Source: Front Plant Sci. 2026 May 13;17:1811600. doi: 10.3389/fpls.2026.1811600 (PMC13212056; doi:10.3389/fpls.2026.1811600)
Supplement: Supplementary file 1 [file SupplementaryFile1.docx]

Table 1 RT-PCR and qRT-PCR primer sequences

| Primer name | Sequences（5’——3’） | Length/bp | Function |
| --- | --- | --- | --- |
| *PpMYB4-Pro*-F | 5’-GGAGGAGAACGAAGTAATGG-3’ | 827 | RT-PCR |
| *PpMYB4-Pro*-R | 5’-GCAGATTTAGGAAGGAGAGG-3’ |  |  |
| *Q-PpMYB4-Pro*-F | 5’-GGGAAGAAGGCTAGGAAGAC-3’ | 144 | qRT-PCR |
| *Q-PpMYB4-Pro*-R | 5’-CTTGACGGACACGGAGTTCG-3’ |  |  |
| *PpMYB4 Qi*-F | 5’-GAAAGCGGTATCAAATGTGC-3’ | 1766 | RT-PCR |
| *PpMYB4 Qi*-R | 5’-AAGATGGGGCTGAAGAGGGG-3’ |  |  |
| *Q-PpUBQ-*F | 5’-AGAAGAAGACCTACACCAAG -3’ | 217 | qRT-PCR |
| *Q-PpUBQ-*R | 5’- GGTTGTAAGGCGTAGGTGAG -3’ |  |  |


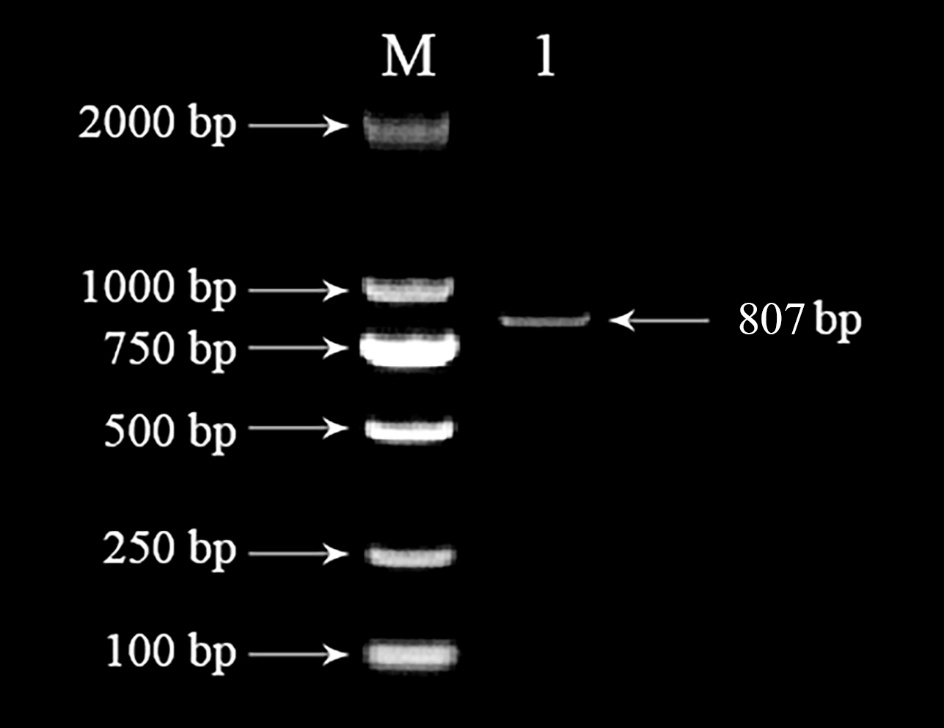


Fig. 1 Electrophoretic analysis of *PpMYB4* amplification in Kentucky bluegrass

Note: M indicates the DL2000 DNA marker; lanes 1-2 indicate the *PpMYB4* PCR products.
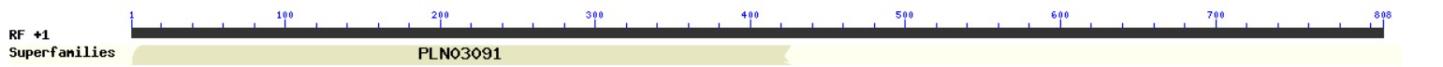


Fig. 2 Conserved amino acid sequence analysis of PpMYB4


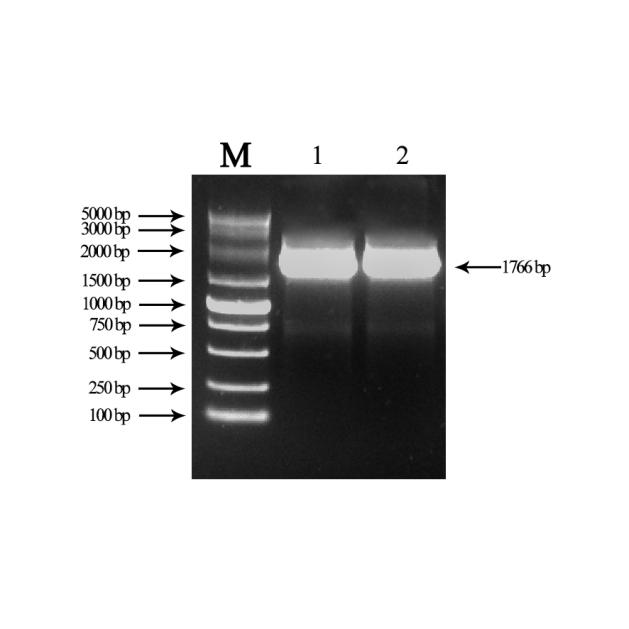


Fig. 3 Electrophoretic analysis of *PpMYB4* promoter


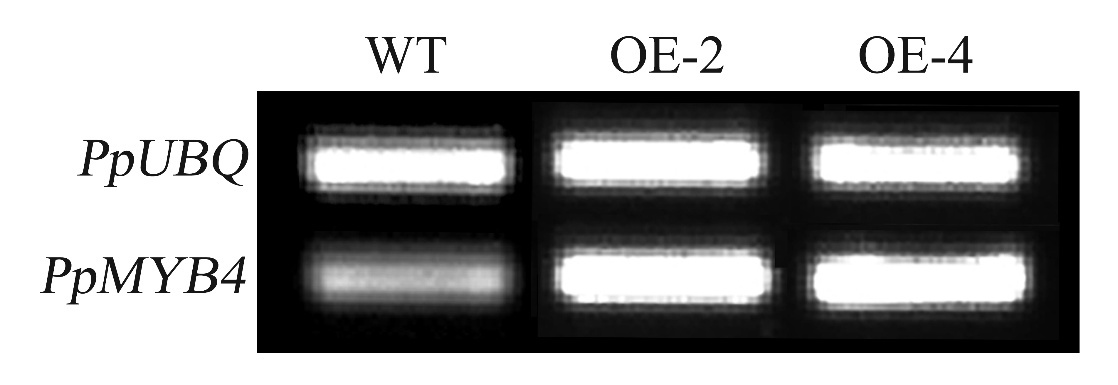


Fig. 4 Expression analysis of *PpMYB4*, using *PpUBQ* as the internal reference gene
